# What Gets Measured Gets Counted: Food, Nutrition, and Hydration Non-Compliance in Ontario Long-Term Care Homes and the Role of Proactive Compliance Inspections, 2024

**DOI:** 10.3390/ijerph22111619

**Published:** 2025-10-23

**Authors:** Kaitlyn R. Wilson, Laura C. Ugwuoke, Sofia Culotta, Lisa Mardlin-Vandewalle, June I. Matthews, Jamie A. Seabrook

**Affiliations:** 1 Brescia School of Food and Nutritional Sciences, Western University, London, ON N6A 3K7, Canada; kwils266@uwo.ca (K.R.W.); sculott@uwo.ca (S.C.); lisamv03@gmail.com (L.M.-V.); 2 Department of Epidemiology & Biostatistics, Western University, London, ON N6G 2M1, Canada; 3 Department of Paediatrics, Western University, London, ON N6A 5W9, Canada; 4 Children’s Health Research Institute, London, ON N6C 2V5, Canada; 5 Lawson Research Institute, London, ON N6A 4V2, Canada; 6 London Health Sciences Centre Research Institute, London, ON N6A 5W9, Canada

**Keywords:** long-term care, legislation, regulations, inspections, food, nutrition, hydration, compliance, Ontario, Canada

## Abstract

Food and nutrition services are critical to the health of long-term care home (LTCH) residents, yet little is known about how regulatory inspections detect non-compliance with Food, Nutrition, and Hydration (FNH) standards. We conducted a cross-sectional study of administrative inspection data from all licensed LTCHs in Ontario, Canada. One inspection report was randomly selected per LTCH, yielding a sample of 623 LTCHs. The data were collected for the period spanning 1 January 2024 to 31 December 2024. The primary exposure was use of the FNH inspection protocol, and the outcome was FNH non-compliance, defined as at least one Written Notification or Compliance Order. Statistical analyses included chi-square tests for categorical variables and independent samples t-tests (including Welch’s t-tests where appropriate) for continuous variables, with effect sizes (Φ, Cramer’s V, Cohen’s d) reported to complement *p*-values. This study did not require research ethics review under Western University policy, consistent with Canada’s Tri-Council Policy Statement (TCPS 2, Article 2.2) regarding use of publicly available data. FNH non-compliance was identified in 12.2% (*n* = 76) of all LTCHs, and in 43.7% of those using the FNH protocol. Use of the FNH protocol was associated with a higher likelihood of detecting FNH non-compliance compared with other inspection protocols (*p* < 0.001, Φ = 0.55). LTCH ownership and inspection type were also associated with detection patterns. This exploratory study provides the first province-wide analysis of FNH non-compliance in Ontario LTCHs. Findings suggest that inspection protocols influence detection of FNH issues, underscoring the need for further comparative and qualitative research to understand the organizational factors underlying non-compliance.

## 1. Introduction

### 1.1. Context of Long-Term Care in Ontario, Canada

Long-term care homes (LTCHs) are healthcare centers that provide 24-h care to individuals whose needs cannot be met in the community [1]. Residents are typically older individuals with complex health conditions and elevated dietary needs [2].

All LTCHs in Ontario, Canada are publicly funded (with contributions from residents who have the means to pay), licensed and regulated by the Ministry of Long-Term Care, and operated under three types of home ownership: for-profit (private sector), non-profit (religious or charitable organizations), or municipal (local government) [3,4]. Every LTCH is regulated under the Fixing Long-Term Care Act, 2021 and its corresponding regulations (Ontario Regulation 246/22) [5,6]. LTC in Ontario is governed by several layers of regulation, each requiring extensive documentation (e.g., mandatory admission, quarterly, annual, and change-of-status clinical assessments; inspections related to public health, fire safety, and occupational health and safety; and requirements concerning workforce training as well as the registration and professional conduct of regulated health professionals). Compliance with the hundreds of requirements in this comprehensive regulatory framework has been identified as a significant challenge [3,7,8]. Indeed, Ontario’s regulatory framework has been perceived as “overly complex, inflexible, and misaligned with the diverse realities of LTCH settings (e.g., urban vs. rural)” [9] (p. 13).

In addition to being subject to mandatory legislation and regulations, LTCHs in Ontario may voluntarily obtain accreditation from a recognized accrediting body [10]. Accreditation offers benefits including a small financial premium from the Ministry of Long-Term Care [11], as well as community prestige and awareness of performance gaps [12]. However, research has shown that this self-regulatory process, which, in Ontario, is funded by the LTCHs themselves, may have limited impact on quality of care [13,14].

A study of long-term care regulations in six countries identified Ontario as one of only two jurisdictions that implement the most deterrence-focused and standardized inspection models aimed at preventing adverse events that jeopardize the safety of residents and staff [15]. The newest legislation (i.e., Fixing Long-Term Care Act) and regulations (i.e., Ontario Regulation 246/22)—upon which current inspections are based—were implemented in response to long-standing concerns raised by residents, staff, family members, advocates, experts, politicians, and researchers, as well as recommendations from Ontario Auditor General reports [16,17,18], Ontario’s Long-Term Care COVID-19 Commission [3], and the Public Inquiry into the Safety and Security of Residents in the Long-Term Care Homes System [7].

Thus, Ontario’s unique and diverse LTCH landscape warrants an exploration into whether type of LTCH ownership and accreditation status are correlated with legislative and regulatory compliance.

### 1.2. Regulatory Oversight Process of LTCHs in Ontario

LTCHs are subject to various inspections by the Ministry of Long-Term Care to determine compliance with legislation and regulations [5,19]. Complaint inspections are conducted in response to concerns raised by residents, staff, family members, or the public [19]. Critical Incident inspections are required when serious events occur, including fires, resident abuse or neglect, sudden or unexpected deaths, prolonged missing residents, outbreaks of communicable diseases, or contamination of the drinking water supply [6,19]. Follow-up inspections involve an inspector revisiting a home to ensure that corrective actions have been implemented by the specified deadline [19]. Proactive Compliance Inspections (PCIs), unannounced annual inspections that last approximately seven days, were introduced in 2021 to replace previous annual inspections that effectively stopped in the year prior to the COVID-19 pandemic [20,21]. Inspection types are not mutually exclusive and can occur in any combination.

Ministry of Long-Term Care inspectors use inspection protocols to guide the inspection process. Inspectors undergo extensive training on the Act and the Regulations, as well as the inspection process and protocols to support uniform application of the standards across all LTCHs. All LTCHs, regardless of their home type, follow the same standards as set by the Fixing Long Term Care Act, 2021 and the Ontario Regulations 246/22. Common protocols include resident care and support services; responsive behaviors; infection prevention and control; pain management; and food, nutrition, and hydration (FNH), among others. Key FNH-related standards reviewed in an inspection include clinical records (e.g., nutrition and hydration assessments, plan of care); food production and storage (e.g., menus for all diets, textures, and fluid consistencies; one portion of every hazardous food served at every meal and snack kept for one week); meal and snack service (e.g., daily and weekly menus posted near the dining room, each resident asked for choice of food and fluids, individual food and fluid intake recorded); and confirmation that staffing requirements for the Registered Dietitian and food service workers have been met [5,6,22].

Inspections of LTCHs are carried out by government inspectors. Public Health Inspectors usually conduct monthly inspections, and, since the onset of the COVID-19 pandemic, they focus primarily on Infection Prevention and Control (e.g., screening, masking, hand hygiene, immunization), while also addressing outbreaks and observing the overall cleanliness of the LTCH. These inspections complement each other but overlap can also occur as both LTC or Public Health Inspectors can write citations if they observe health and safety issues during their inspections (e.g., evidence of rodent infestation, improper temperatures in vaccine fridges, residents being fed snacks by employees who are not wearing gloves, food not covered).

When non-compliance is identified, inspectors may issue Written Notifications, Compliance Orders, and/or Administrative Monetary Penalties [5,21]. Written Notifications are issued when a violation does not pose an immediate risk to residents. Compliance Orders are more serious and require specific corrective actions within a set timeframe, often followed by re-inspection. Administrative Monetary Penalties are financial penalties applied in cases of repeated or high-risk non-compliance, intended to deter further violations and reinforce accountability [5,21]. Following an inspection, inspectors release a publicly available report that outlines information about the LTCH, the type of inspection, the findings observed, and any enforcement actions or sanctions issued [5,19].

Some studies have investigated general compliance patterns in LTCHs. For example, de Witt et al. [23] analyzed incidents related to responsive behaviors documented in Ontario’s public inspection reports to determine compliance with related legislation. Crea-Arsenio et al. [24] analyzed inspection reports related to neglect. Mashouri et al. [25] investigated whether quality indicators could predict future inspection performance in Ontario LTCHs. Other researchers evaluated the effectiveness of enforcement mechanisms to identify gaps in regulatory frameworks [26] or examined whether legislation in Ontario effectively protects residents from abuse [27]. In non-Canadian contexts, studies have also explored how facility characteristics, such as size, ownership, and location, relate to non-compliance notices or sanctions during inspections [28,29,30]. To the researchers’ knowledge, neither the prevalence of non-compliance with FNH-related standards in Ontario LTCHs nor the characteristics associated with non-compliance findings have been investigated.

### 1.3. Significance of Food, Nutrition, and Hydration in LTCHs

The importance of complying with FNH standards in LTCHs cannot be overstated. The high prevalence of chronic illnesses and multiple co-morbidities in a population largely composed of older adults significantly increases the risk of foodborne illness. In such vulnerable groups, lapses in food safety can result in severe and potentially fatal consequences [31,32]. For instance, the 1985 salmonella outbreak at a nursing home in Ontario, described as “the most serious epidemic of its kind in North American institutional history” [33] (p. 8), resulted in 69 cases of hemorrhagic diarrhea, 19 resident deaths, and widespread public alarm [33]. Inspection findings revealed numerous food safety violations, as well as the failure of the LTCH to report the outbreak to the local Medical Officer of Health [33].

Sadly, issues continue to arise in Ontario LTCHs. Between January 2018 and May 2019, over 660 food- and nutrition-related incidents were reported, including 27 unexpected deaths due to aspiration or choking, and over 1100 avoidable emergency room visits related to food and fluid intake [16]. During the COVID-19 pandemic, Canadian Armed Forces personnel had to provide care and medical services to residents living in shocking conditions in five Ontario LTCHs [34].

Even outside of a pandemic, the prevalence of nutrition- and hydration-related conditions in LTCH is disturbingly high. Malnutrition ranges from 44–62% [31,35], 19% of residents experience dehydration [36], 24% have dysphagia [37], and 19% suffer from pressure injuries [36]. In addition to increased health care costs [38], consequences of nutrition-related conditions range from increased risk of falls, infections, and pressure injury to a three- to six-fold increase in mortality [31]. Most of these conditions are preventable and can be managed through well-planned menus, comprehensive dietary assessments, relationship-centered dining, and compliance with FNH standards [36,39,40]. The gap, however, is that there has been no research published on non-compliance with FHN regulations.

Given the significant impact of FNH on residents’ health and quality of life, the aim of the present study is to analyze LTC inspection reports to provide a deeper understanding of the extent and dimensions of FNH-related issues in Ontario LTCHs. The research objectives for this study are:

1. Describe the prevalence and distribution of FNH-related non-compliance under the Fixing Long-Term Care Act, 2021 and Ontario Regulation 246/22.
2. Explore whether non-compliances are associated with selected LTCHs and inspection characteristics.

This research will contribute to our understanding of FNH-related issues in LTCHs and provide guidance to dietitians and other LTC staff to help them not only meet government regulations but also provide quality care to residents.

## 2. Materials and Methods

### 2.1. Study Design

This retrospective study investigated Ministry of Long-Term Care inspection reports of LTCHs in Ontario, Canada. This study did not require research ethics review under Western University Policy 5.1 Research Exempt from Research Ethics Approval, Section 5.1.1.2, consistent with Canada’s Tri-Council Policy Statement (TCPS 2, Article 2.2) regarding use of publicly available data.

### 2.2. Inclusion Criteria

Inspection reports are mandatorily published by the Ontario Ministry of Long-Term Care and were accessed through its publicly available website [19]. To ensure that all LTCHs were represented, one inspection report was randomly selected per home from those available during the study period (1 January 2024 to 31 December 2024) and downloaded for analysis. One inspection report per LTCH was randomly selected to preserve independence of observations and to avoid over-representing homes with multiple inspections in the same year. This approach provides a province-wide snapshot of compliance while minimizing clustering effects, though it does not capture repeated patterns of non-compliance within individual homes.

Data were extracted by trained graduate students using a consistent procedure that was developed iteratively by the research team. Specifically, inspection reports were reviewed in detail, and an Excel spreadsheet was used to record each cited section of the Act or the Regulations, with new columns added as required. Although we did not employ dual review or κ agreement due to the large volume of records and resource constraints, coding decisions were discussed among the team to ensure consistency. These steps helped maintain reliability and transparency within the scope of the project.

### 2.3. Independent Variables

Independent variables (ownership, accreditation status, geographic location, and bed count) were selected based on prior literature and regulatory relevance. Inspection-level characteristics, including type (complaint, critical incident, follow-up, proactive compliance inspection), inspection length, number of on-site days, number of protocols used, season, and use of the FNH protocol, were included to capture inspection intensity and procedural variation. This selection enabled us to examine how both structural facility characteristics and inspection processes were associated with the detection of FNH non-compliance.

### 2.4. Outcome Variable

The outcome was the presence of at least one FNH-related non-compliance, defined as either a Written Notification or a Compliance Order. Written Notifications and Compliance Orders were combined because both represent enforceable findings of non-compliance, and Compliance Orders were relatively rare, making stratified analysis infeasible. Administrative Monetary Penalties were excluded because they were rarely applied and were always issued in relation to an underlying Written Notification or Compliance Order. Their inclusion would therefore not alter the classification of LTCHs as non-compliant.

### 2.5. Database Description

All data were compiled into an Excel spreadsheet between December 2024 and April 2025, and verified for accuracy in May 2025. Each finding of non-compliance was assigned to its respective section/sub-section of the Fixing Long-Term Care Act [5] or Ontario Regulation 246/22 [6]. To enhance clarity and comprehension, the 239 sections and sub-sections that had non-compliances were organized into 13 categories (Table S1). There was only one point of missing data, for number of beds. Thus, this LTCH was excluded only for analyses that directly involved this variable.

### 2.6. Statistical Analyses

All analyses were conducted using IBM SPSS Statistics version 30.0 (IBM Corp. Armonk, NY, USA). Descriptive statistics were reported as means and standard deviations for continuous variables, and as frequencies and percentages for categorical variables.

Independent samples t-tests were used to compare means of continuous variables between two groups of FNH non-compliance: (i) no FNH non-compliance, (ii) at least one FNH non-compliance. Welch’s t-tests were applied when Levene’s test indicated unequal variances. Effect sizes for t-tests were reported using Cohen’s d, with values of 0.20, 0.50, and 0.80 interpreted as small, medium, and large, respectively [41,42].

Pearson chi-square tests were used to assess associations between categorical independent variables and FNH non-compliance. Effect sizes were reported as Phi (Φ) for 2 × 2 tables and Cramér’s V for larger tables, with thresholds of 0.10, 0.30, and 0.50 interpreted as small, moderate, and large effect sizes, respectively [41,42]. Standardized residuals were used to identify statistically significant cells within contingency tables, with absolute values > 1.96 indicating higher- or lower-than-expected frequencies.

Statistical significance was defined as a *p*-value of <0.05. Missing data were minimal: one LTCH lacked bed count information and was excluded only from analyses directly involving that variable. This study was designed as a descriptive, cross-sectional analysis to characterize inspection patterns rather than to estimate independent predictors; therefore, multivariable regression was not performed, and findings are interpreted as associative and descriptive.

## 3. Results

### 3.1. Sample Description

At the time of data analysis, there were 675 LTCHs in Ontario listed on the public reporting website [19]. Of these, 623 LTCHs had at least one published inspection report from January 2024 to December 2024. Two descriptor variables were collected to contextualize our sample and better understand the scope of PCIs in 2024: (1) the total number of inspections per LTCH in 2024 (continuous), and (2) whether a PCI was conducted at the LTCH in 2024 (binary: yes/no). There were 2401 inspections in this timeframe. On average, each LTCH underwent 3.9 inspections of any type (SD = 1.7). In 2024, less than half (43.7%, *n* = 272) of the 623 LTCHs had at least one PCI, representing 11.3% of total inspections.

One inspection report was randomly selected per LTCH for analysis (*n* = 623), representing 25.9% of all inspections conducted in 2024. Among the included LTCH, there were a total of 80,340 licensed beds, with an average of 129.2 beds per home (SD = 75.0).

Table 1 summarizes the LTCH- and inspection-level characteristics of the sample. Most LTCHs (82.2%) were accredited, 55.1% were for-profit, and 71.9% were located in an urban area. The total number of inspection days across all randomly selected reports was 3678, with an average of 5.9 days per inspection (SD = 3.5). Of these, 3387 days (92.1%) were conducted on-site, while 291 (7.9%) were conducted off-site. Per report, there was an average of 5.4 on-site inspection days (SD = 3.2) and 0.5 off-site inspection days (SD = 1.9). Within the sample, inspectors used 23 separate inspection protocols to check for regulatory compliance (Table S2). Inspectors used an average of 4.5 protocols per inspection (SD = 3.3). Approximately one-quarter (24.2%) of the sampled inspections used the FNH inspection protocol. The majority of inspections (75.4%) were triggered by a critical incident. Only 11.2% of the sampled reports were a PCI.

### 3.2. Non-Compliance Prevalence and Distribution

A total of 2183 non-compliances were identified across the sampled reports. Of these, 1939 (88.9%) were Written Notifications and 243 (11.1%) were Compliance Orders. On average, each report cited 3.5 non-compliances (SD = 4.3). The median number of non-compliances per report was 2.0. Of the 623 reports analyzed, a total of 148 (23.9%) had no non-compliance findings in any category.

**Figure 1:**
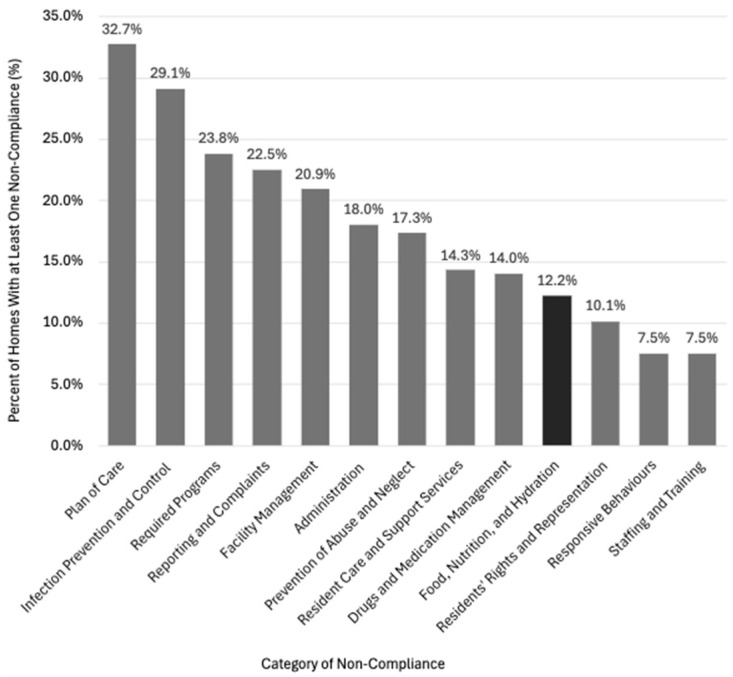
Percentage of homes with at least one non-compliance in each category, based on sampled inspection reports (*n* = 623).

FNH-related non-compliance was identified in 76 reports (12.2%), with a range of 1–5 of these findings per report (Figure 1). These 76 reports accounted for 120 FNH-related non-compliances—5.5% of all 2183 non-compliances identified. Of FNH non-compliances, 111 (92.5%) were Written Notifications and 9 (7.5%) were Compliance Orders. Most FNH-related non-compliances (*n* = 105, 87.5%) pertained to dining and snack service, menu planning, food production, and nutritional care and hydration programs. The remaining 15 non-compliance findings (12.5%) involved areas such as dietary services, staffing (e.g., dietitian, food service workers), care planning, and monitoring of weight changes.

### 3.3. Correlates of Food, Nutrition, and Hydration Non-Compliance

Table 2 summarizes the results of independent samples t-tests comparing continuous LTCH and inspection characteristics between two groups: LTCHs with no FNH non-compliance (*n* = 547) and those with at least one FNH non-compliance (*n* = 76). LTCH with at least one FNH non-compliance finding had longer inspections than homes with no FNH non-compliance findings (M = 8.9 days, SD = 3.1 vs. M = 5.5 days, SD = 3.4; *p* < 0.001, d = 0.98, respectively). Specifically, LTCHs with at least one FNH non-compliance were associated with more on-site inspection days (M = 8.3, SD = 2.9) compared to LTCHs without FNH non-compliance (M = 5.0, SD = 3.4, *p* < 0.001, d = 1.08). Likewise, there was a significant difference in the number of inspection protocols used between homes with no FNH findings (M = 3.9, SD = 2.7) compared to LTCHs with at least one non-compliance finding (M = 8.8, SD = 3.6), *p* < 0.001, d = 1.70. There was no statistically significant difference in the number of beds or number of off-site inspection days between the two groups of non-compliance.

Table 3 presents the results of Pearson chi-square tests examining associations between categorical independent variables and the presence of at least one FNH non-compliance. While the overall chi-square test reached borderline statistical significance for the LTCH ownership variable (*p* = 0.04), the effect size was small, suggesting limited practical importance. No other LTCH characteristics were associated with FNH non-compliance. In terms of inspection characteristics, at least one FNH non-compliance finding was associated with critical incident inspections (43.4%), compared to non-critical incident reports (56.6%, *p* < 0.001, Φ = 0.28). Of LTCHs with at least one non-compliance, more reports were PCI (52.6%) compared to non-PCI (47.4%, *p* < 0.001, Φ = 0.28). Specifically, of all PCI inspections (*n* = 70), 40 LTCHs (57.1%) cited at least one FNH non-compliance.

In addition, of LTCHs that had at least one non-compliance, there were more inspections that used the FNH inspection protocol (86.8%) compared to those that did not use the protocol (13.2%, *p* < 0.001, Φ = 0.55). Specifically, of inspections that used the FNH protocol (*n* = 151), 66 LTCH (43.7%) had at least one FNH non-compliance.

The observed association between FNH protocol use and higher detection of non-compliance should be interpreted as descriptive, reflecting differences in inspection practices rather than a causal effect of the protocol itself.

## 4. Discussion

### 4.1. Main Findings

This is the first study to explore non-compliance with legislation [5] and regulations [6] related to FNH using publicly available reports of LTCH inspections in Ontario, Canada. These reports are important because they are used by the provincial government to monitor performance of LTCHs, and by the public to judge the suitability of LTCH options for themselves or their loved ones. Our retrospective analysis of 623 randomly selected inspection reports revealed that non-compliance with FNH standards was documented in relatively few inspectors’ visits. However, a key contribution to the literature is our finding that the use of the FNH inspection protocol by inspectors was strongly associated with the detection of at least one FNH-related non-compliance. This suggests that more intentional inspections, such as PCI, may uncover issues related to FNH.

Non-compliance with food, nutrition, and hydration standards has direct implications for resident well-being, including risks of malnutrition, dehydration, and infection. By mapping inspection characteristics linked to detection patterns, our findings help policymakers understand how inspection design and intensity influence surveillance sensitivity. These insights can guide more equitable and effective monitoring strategies to safeguard resident nutrition and hydration in long-term care.

### 4.2. Significance of Inspection Characteristics and FNH Non-Compliance

The seemingly low rate of non-compliance with FNH standards (5.5% of all non-compliances in our sample) could be due to the types of inspections that are conducted. During complaint, critical incident, and follow-up inspections, inspectors do not typically assess FNH compliance unless it directly relates to the issue at hand. For example, complaint-triggered inspections means that complaints are driving when, where, and what to inspect [26,47]. However, low or no complaints do not necessarily reflect low risk or high compliance with standards [3]. Importantly, three-quarters of inspections in our sample were initiated in response to critical incidents; however, many critical incidents that trigger inspections are unrelated to FNH (see [6], s. 115). This is evident in our finding that critical incident inspections were associated with a lower prevalence of FNH non-compliance detection. Key informant interviews with individuals in the Ontario LTC sector also revealed that most inspections are triggered either by complaints from residents or their family members, or by critical incidents reported by LTCHs [26]. Participants perceived that inspections only identify a portion of incidents related to non-compliance, and the potential for detecting non-compliances is low in the absence of more inspections [26]. Rather than more inspections overall, our analysis suggests that PCI in particular may be more effective for detecting non-compliance related to FNH in LTCHs.

PCI are the only type of inspection that consistently examines FNH non-compliance at each visit. Therefore, narrowing our focus to the prevalence of FNH-related non-compliance in PCI provided insight into which type of inspection is more likely to result in the detection of this type of non-compliance. Concerningly, half (57.1%) of our sample’s PCI reports detected at least one non-compliance related to FNH standards. This suggests potentially high levels of non-compliance with FNH standards, which could pose a risk to residents’ health and well-being. Our research thus reinforces the need for the full rollout of the PCI program. A PCI utilizes a standardized set of inspection protocols, including the FNH inspection protocol [48]. Once fully adopted, this standardized approach may help improve both consistency and comprehensiveness of inspection findings.

This study also revealed a strong positive association between inspection length in days and detection of FNH non-compliance, suggesting that thorough inspections are positively correlated with more non-compliance citations. The investigation into on- vs. off-site inspections was important, as the effectiveness of remote inspections has been described as “questionable” [3] (p. 165), a concern echoed by inspectors themselves [49]. In our sample, there was a strong positive correlation between on-site days and the detection of FNH non-compliance. In contrast, off-site inspection days showed no significant association with detection. This result could be attributed to a lack of effectiveness of remote inspections, or to inspectors not often actively assessing FNH non-compliance during remote inspections. Further analysis into whether remote inspections are associated with total non-compliances, and not just FNH, is warranted. Given that remote/off-site inspections were not associated with detection of non-compliance, it is recommended that inspections be conducted in person rather than relying on verbal reports from staff. Exceptions to this would be, for example, during a viral pandemic when personal protective equipment may be in short supply (as was the case at the beginning of COVID-19). In that instance, it would be safer for residents, staff, and inspectors if inspectors remotely check in to obtain updates (e.g., number of infections, staff immunization), rather than running the risk of transmitting the virus between inspection sites.

A key finding was that the FNH Inspection Protocol was strongly associated with the detection of at least one related non-compliance, suggesting that non-compliance is detected when FNH areas are examined. This may be explained by increased measurement intensity. There was also a strong association between the number of inspection protocols used and the detection of FNH non-compliance, which could suggest that the ability of inspectors to detect FNH-related non-compliance is not overshadowed when other home areas are examined alongside FNH. Evaluating the cost-effectiveness of Ontario’s current inspection processes, specifically how inspection time and intensity translate into meaningful findings that can support improved care and system-level accountability within LTC, is warranted.

### 4.3. No Significant Association Between LTCH Characteristics and FNH Non-Compliance

No individual LTCH characteristic was significantly associated with the presence of at least one or more FNH non-compliance findings. Two key characteristics—accreditation and LTCH ownership—are discussed here.

The lack of association between LTCH accreditation status and FNH non-compliance reinforces previously identified concerns that accreditation in and of itself may not translate into higher quality care [13,14,50]. This finding may suggest little value in LTCHs paying for this status (even with the MLTC providing some cost recovery), as non-significant differences in FNH non-compliance are contrary to the purpose of accreditation (i.e., that it is an indicator of higher quality standards). Thus, this audit may not be a useful or productive investment (in time, money, or effort). This is relevant in terms of resource allocation as both the LTCHs and the Ministry of Long-Term Care could realize cost savings by not paying for it. Canadian researchers have recommended that infectious disease outbreaks could be best managed by inspections conducted by relevant public health units rather than an accreditation body [51].

Accreditation potentially weakens the value of enforcement as it gives the impression (to several audiences) that the LTCH is meeting legislative standards; however, the accreditation process does not assess whether laws or regulations are being followed. That is the purview of the Ministry of Long-Term Care Inspections Branch through the inspection process that was the focus of this study. Accreditors document whether policies exist. They do not assess whether those policies are being implemented. Thus, eliminating the extensive preparation of documentation in advance of an accreditation visit would free up valuable staff time that could be better invested in providing care and meeting the detailed legislative requirements already in place.

As noted previously, foodborne illness outbreaks can be just as fatal as other infectious diseases [33]; therefore, consistent and effective regulatory oversight of FNH is imperative. It has been recommended that the Ministry of Long-Term Care, the Ministry of Labour, Training and Skills Development, and public health units increase coordination to facilitate comprehensive and effective inspections [3,49]. Food safety inspections of Ontario LTCH kitchens are conducted by Public Health Inspectors independent of LTC inspections, potentially creating gaps in regulatory oversight, where responsibilities are either duplicated or overlooked. This can result in inefficient use of resources and an increased risk to residents. Our findings suggest that PCIs were conducted in less than half of all LTCHs in 2024, the only inspection type that consistently analyzes compliance with FNH regulatory standards at every visit. As such, our findings also support the recommendation of increased coordination amongst these three government bodies.

Our recommendations to reduce documentation burden and expand protocol use may seem contradictory; however, reducing documentation burden relates to staff in LTCHs. Expanding protocol use, on the other hand, refers to work completed by LTC Inspectors. Elimination of the accreditation process would reduce documentation burden. Expanding protocol use may result in greater detection of non-compliance related to FHN, but it does not automatically translate into more documentation for the staff. For example, non-compliance noted by an Inspector regarding Personal Support Workers “simultaneously assisting more than two residents who need total assistance with eating or drinking” [Ontario Regulation 246/22 Sec. 79(2)a] does not add to the documentation burden experienced by staff. Implementing a risk-based, targeted protocol strategy by identifying high-risk FNH items, while reducing the volume of low-value documentation, would increase efficiency and effectiveness.

In Ontario, the literature on various dimensions of quality performance in LTCHs by ownership type presents mixed findings. In our sample, the lack of association between home ownership and FNH non-compliance suggests that for-profit, non-profit, and municipal homes have similar FNH non-compliance prevalence. In contrast, Baumann et al. [27] suggest that for-profit LTCHs in Ontario had a greater case percentage of abuse cited in inspection reports than non-profit or municipal homes, although they did not account for other variables. de Witt et al. [23] suggest that for-profit LTCHs in Ontario did not perform as well as non-profit LTCHs in terms of documented responsive behaviors in inspection reports, but this result was not consistent across geographic regions. In contrast, Crea-Arsenio et al. [36] found that for-profit LTCHs performed better than non-profit homes in several areas. For example, the likelihood of pressure ulcers was 54% higher in municipal LTCHs and 21% higher in non-profit homes than in LTCHs with private ownership. The risk of dehydration was also twice as high in municipal LTCHs and 15% higher in non-profit LTCHs than in for-profit LTCHs [36].

Similar to our findings, other research shows no significant differences between LTCHs by profit status. For example, relationship-centered care, which has been identified as a factor linked to quality of life for LTCH residents and their families [52], was not significantly associated with for-profit ownership in either general or dementia units [53]. In another study, LTCH characteristics such as age of building, home size, and geographic location explained the largest variance in relationship-centered care and task-focused practices during mealtimes; however, LTCH ownership status was not associated with these types of mealtime practices [54]. Encouraging relationship-centered dining, particularly with family members, is a valuable strategy to enhance residents’ dining experiences [55,56]. However, even if residents have family members who are willing and able to participate in mealtimes, it is uncertain if they are aware of FNH standards (e.g., feeding a loved one while seated, or serving only foods and beverages indicated in the care plan). A lack of awareness may inadvertently contribute to increased findings of non-compliance. Dietitians have long expressed frustration regarding insufficient time to conduct proactive education that would enhance care [57].

Ontario’s LTC regulatory system is standardized, complex, and deterrence based, which is in line with other jurisdictions where for-profit ownership is high (e.g., US, England) [15]. While increased use of the FNH protocol may reflect stronger regulatory enforcement, increasing the regulation of structural indicators (e.g., level, intensity, and training of staff) [15] would have a greater impact on the quality of FNH and other areas of care.

### 4.4. Challenges and Recommendations

Complex barriers within LTCHs pose challenges to the implementation of all LTC standards. In their qualitative evidence synthesis, McArthur et al. [58] identified key barriers (e.g., staff, time, financial) and facilitators (e.g., teamwork and organizational support) to implementing evidence-based practice guidelines in LTC. These same challenges have been documented repeatedly with regard to LTC regulations [3,7,8,9,59]. One of the most important challenges in LTC is the burdensome regulatory process, which is widely perceived as adversarial and has been linked to reduced staff morale and feelings of fear and distress [7,9]. For example, it is not uncommon for staff members to feel anxious or overwhelmed when an inspector stands nearby taking notes while they attempt to plate meals that meet the correct portion sizes and texture modification for dozens of residents—each with an individualized care plan—under time pressure during a busy mealtime.

As Banerjee and Armstrong [8] explained, each regulation makes sense on its own (e.g., texture-modified diets for residents with swallowing difficulties); however, the sheer number of regulations is burdensome, and the required documentation takes time away from actual care [8]. This ‘culture of documentation’ is exacerbated by insufficient staffing—one of the core problems in the sector [3,16,18,60,61,62]—yet research has shown that adequate staffing is positively associated with quality of nutrition care and residents’ quality of life [56,63,64].

Malnutrition and dehydration have been strongly associated with increased frailty, impaired immune response, delayed recovery, and higher mortality rates among older adults in institutional settings [65,66]. Additionally, inadequate FNH support contributes to pressure injury development and poor management of chronic conditions, further burdening care systems [31,38]. Accountability for meeting FNH legislative and regulatory standards is one aspect of ensuring quality FNH care, but accountability is not solely the responsibility of employees. While workers should be accountable for public expenditures, the state should also be accountable to workers, ensuring sufficient resources to provide good care [8].

As recommended by Justice Gillese [7], the Ministry of Long-Term Care has implemented a new Investigations Unit which will not only enhance enforcement and accountability through reactive measures, but also act as a “proactive deterrent to improve compliance and ensure resident safety” [67] (p. 1). If regulators only use pyramids of sanctions—a regulatory approach that focuses primarily on escalating penalties for non-compliance—then they risk only focusing on the poor performers and missing opportunities to support a positive trend in compliance [26]. Thus, the Ministry’s new compliance continuum, where education and outreach accompany mandatory reporting through a cycle of guiding, communicating, measuring, reporting, and adapting [21] is a promising step forward and may prevent non-compliances related to FNH.

Given the complex challenges in this healthcare sector, both LTC workers and inspectors deserve our appreciation. As Justice Gillese explained, the system is not broken, but rather one that must build on its strengths while acknowledging its vulnerabilities [7]. Recommendations for improvement in LTC have been identified in more than 80 reports published from 1998–2020 at an estimated cost of over 23 million dollars [61]. Our research makes a modest contribution to this extensive body of work. Drawing on both prior literature and our study’s findings, we propose three recommendations: (1) enhance regulatory compliance; (2) focus on structural changes to improve food and nutrition care; and (3) promote greater appreciation of the LTC sector.

#### 4.4.1. Enhance Regulatory Compliance

While some activities are justifiably tightly controlled through regulations (e.g., medications, food safety), others could be more open to interpretation (e.g., presentation, timing, and availability of food) [68]. Justice Gillese recommended that more weight be given to findings of non-compliance associated with high risk (e.g., failure to report suspected abuse or neglect) than non-priority issues such as failing to ensure that planned menu items are available at each meal and snack [7]. Our analysis highlights that what gets measured gets counted; however, shifting from a reductionist approach [7,8] to a more just and less punitive culture [7] may enhance workers’ capacity to comply. In addition, transparency of the inspection process and clarity of measures are as important as non-compliance, and both could reduce negative emotions, improve staff morale, increase motivation to meet standards, and decrease the risk of public fear about LTCHs.

Future research could explore potential revisions to the quantity and prescriptive nature of regulations to enhance compliance. For example, reducing documentation burdens—such as re-evaluating the requirement to retain a record of every menu substitution for at least one year—could support a more flexible and pragmatic approach to implementation. Such changes may ease pressure on staff [9], better align with residents’ individualized needs [9], and simplify the inspection process for regulatory officials.

#### 4.4.2. Focus on Structural Changes to Improve Food and Nutrition Care

Several studies have recommended strategies to improve FNH care in LTC [39,40,69]. Keller et al. [56] proposed specific interventions related to nutrition care, food quality, eating assistance, and mealtime experiences, and rated them based on their feasibility and the entities that would be accountable for implementation [56]. Their ratings assumed adequate staffing [56]; thus, this foundational requirement would need to be met for these FNH-related practices to become reality. As Pat Armstrong recommended: “Long-term care reforms should focus on structural changes such as staffing levels, rather than regulations designed to control the actions of staff” [70] (p. 1).

Registered dietitians bring expertise in assessing nutritional risk, developing individualized care plans, and coordinating nutrition interventions for conditions such as malnutrition, dysphagia, and chronic disease [71]. Research suggests that when dietitians are actively engaged in resident care and supported by a well-trained interdisciplinary team, measurable improvements occur in food intake, resident satisfaction, and health outcomes [56,72]. On the other hand, nutrition managers, cooks, and dietary aides directly influence mealtime quality, safety, and satisfaction [73,74,75]. Dietary workers also provide critical ‘social care’—engaging in conversations, listening, and offering companionship—that is difficult to quantify but deeply valued by residents [75].

The Ministry of Long-Term Care in Ontario has committed to investing up to $4.9 billion dollars to hire and retain thousands of direct care staff, as well as providing a 20 percent increase in direct care time provided by allied health professionals such as dietitians [67]. They have also doubled the number of inspectors [76], a move reflecting an emphasis on regulatory enforcement. These dual approaches reflect the Ministry’s ongoing attempt to balance investments in structural improvements with regulatory oversight.

#### 4.4.3. Promote Greater Appreciation of the LTC Sector

Increasing positive recognition for, and placing higher value on, the work done by thousands of dedicated management and staff in LTC [9] might reduce negative feelings that compromise the ability of workers to comply with regulations. A powerful example of how top-down oversight can undermine morale and reduce capacity to comply with regulations is represented in this comment from a frontline worker in LTC: “We have both Ministry of Health and Public Health at the same time…it was so insulting and so degrading to have outside people coming in to teach us what to do like we had never been in the sector before” [9] (p. 9). This experience would be particularly upsetting for experienced employees interacting with an inspector newly graduated from university. Thus, to balance the “what gets measured gets counted” framework, we call for meaningful, respectful oversight paired with real investment in the sector.

### 4.5. Strengths and Limitations

This is the first study in the Canadian context to explore non-compliance related to FNH under the Fixing Long-Term Care Act, 2021 [5] and its associated regulation [6]. Not only was information revealed about the prevalence of non-compliance, but associations with other variables were also assessed. These findings contribute to a greater understanding of FNH in Ontario LTCHs, as well as the modalities that detect non-compliance. Our sample represents all 623 LTCHs operating in Ontario in 2024. Thus, we are confident that the findings can be generalized to all LTC inspections during that year. Since PCI are annual inspections, and if we assume that no LTCH had more than one PCI in 2024, then the number of PCIs conducted in 2024 (*n* = 272) represents 11.3% of total inspections (*n* = 2401). This closely represents the LTCH characteristic in our sample (11.2%, *n* = 70). The mix of LTCH ownership in our sample also reflects the LTCH landscape in Ontario (i.e., 58% for-profit, 24% non-profit, 16% municipal) [3]. The percentage of Written Notifications in our sample is close to the percentage of Written Notifications reported by the MLTC from April to December 2022 (88.9% vs. 79.3%, respectively) [21]. Examining all types of inspections allowed the exploration of associations between inspection type and FNH non-compliance findings. Future research can focus on PCI only, as this standardized inspection approach may offer better comparisons between LTCHs. Analyzing inspection reports published in 2024, the first full year following the end of COVID-19 as a global health emergency [77], represented a timeframe less influenced by that tragic era.

The findings in this study are also subject to limitations. First, although the use of publicly available reports is supported in the literature [23,24], inspection reports are a secondary source of data, as inspectors analyze primary sources of information, then generate and disseminate a public version that is posted on the MLTC website. Thus, there may be discrepancies between these two sources. Second, due to the interrelated nature of FNH services with other aspects of residents’ care, it is possible that FNH non-compliance was recorded under other sections of the regulations and thus underestimated in these results. This phenomenon would reflect the findings of de Witt et al. [23], where incidents of non-compliance related to responsive behaviors were cited under unexpected sections of the regulations. In our sample, it is possible that “Plan of Care” could include assessments related to FNH, and “Infection Prevention and Control” could include hygiene measures for the prevention of foodborne illness.

We relied on one inspection report per LTCH, which does not capture variability in compliance across multiple inspections or over time. While this approach avoided statistical dependence between observations, it may have under- or over-estimated the true prevalence of non-compliance in individual homes. Future research using multi-year or multi-inspection designs would provide a more comprehensive picture. Future research could include qualitative analysis of inspection reports to better understand the scope and contextual drivers of FNH non-compliance in LTCHs and whether non-compliances were high-, medium-, or low-risk. More advanced quantitative analyses could also reveal whether LTCH characteristics are associated with all non-compliance findings in Ontario LTCHs and not just FNH. Future research could also explicitly compare Canadian LTCH ownership and quality patterns with those observed internationally.

Our outcome measure combined Written Notifications and Compliance Orders, which limited our ability to differentiate between lower- and higher-severity findings. We also excluded Administrative Monetary Penalties due to their rarity and delayed timing, which restricts comparability with inspection-based outcomes.

Because this was a cross-sectional, observational study based on administrative inspection data, findings should be interpreted as associations rather than causal relationships. In particular, the higher detection of non-compliance when the FNH protocol was used may reflect greater inspection intensity or selection of homes already considered at higher risk, rather than an effect of the protocol itself. Detection and selection bias remain important limitations that should be considered when interpreting these results.

Our analyses were exploratory and descriptive, intended to provide a province-wide snapshot of FNH non-compliance. Future research could build on these findings by applying multivariable regression or count models to disentangle the independent contributions of ownership, inspection type, and other facility characteristics.

Finally, although this study used quantitative data from the inspection reports, a separate qualitative study could provide important insights into why non-compliance occurs. Integrating qualitative and quantitative approaches would strengthen understanding of the factors driving compliance.

## 5. Conclusions

This study contributes new evidence to the under-researched area of FNH non-compliance in LTC and the factors that contribute to non-compliance findings documented in inspection reports. FNH protocol use was associated with higher detection of non-compliance, but this pattern should be understood as descriptive and reflective of inspection practices, not as evidence of a causal effect. These findings have relevance for regulatory planning and public-health monitoring. These results may also help to improve the inspection process, guide resource allocation, and support policy and practice decisions aimed at strengthening FNH-related care in Ontario’s long-term care sector. This is important as demand for LTC grows and incoming residents present with increasingly complex needs. Future research could incorporate both quantitative and qualitative analyses of LTCH inspection reports to better understand factors contributing to non-compliance and to inform recommendations that help all stakeholders strengthen LTC for residents, families, staff, and inspectors.

## Figures and Tables

**Table 1:** Characteristics of Ontario long-term care homes and associated inspection reports published in 2024 (*n* = 623).

Variables	*n* (%)	Mean (±SD)
LTCH Characteristics		
Number of beds		129.2 (75.0)
Accredited	512 (82.2)	
For-profit	343 (55.1)	
Non-profit	173 (27.8)	
Municipal	107 (17.2)	
Urban	448 (71.9)	
Rural	175 (28.1)	
Inspection Characteristics		
Inspection length (days)		5.9 (3.5)
On-site inspection days		5.4 (3.2)
Off-site inspection days		0.5 (1.9)
Number of protocols used		4.5 (3.3)
Complaint inspection	231 (37.1)	
Critical incident inspection	470 (75.4)	
Follow-up inspection	119 (19.1)	
PCI	70 (11.2)	
Other type of report	24 (3.9)	
Used the FNH Inspection Protocol	151 (24.2)	
Spring	147 (23.6)	
Summer	159 (25.5)	
Fall	203 (32.6)	
Winter	114 (18.3)	

LTCH, long-term care home; SD, standard deviation; PCI, Proactive Compliance Inspection; FNH, food nutrition and hydration. Urban: city/town with >30,000 people or within a 30-min drive from such a location [43]. Driving distances from urban centers were calculated from Google Maps [44] and population data were drawn from Canada’s 2021 Census [45]. Spring: 20 March–19 June; Summer: 20 June–21 September; Fall: 22 September–20 December; Winter: 21 December–19 March [46]. Continuous variables are reported as mean ± standard deviation, categorical variables as percentages. Inspection types are not mutually exclusive and can occur in any combination.

**Table 2:** Comparison of continuous LTCH and inspection characteristics with FNH non-compliance in Ontario Long-Term Care Homes in 2024 (*n* = 623).

Continuous Characteristics	No FNH Non-Compliance (*n* = 547) Mean (±SD)	≥1 FNH Non-Compliance (*n* = 76) Mean (±SD)	*p*-Value	Effect Size (d)
LTCH Characteristics				
Number of beds	128.9 (74.1)	131 (82.0)	0.79	-
Inspection Characteristics				
Inspection length (days)	5.5 (3.4)	8.9 (3.1)	<0.001	0.98
On-site inspection days	5.0 (3.0)	8.3 (2.9)	<0.001	1.08
Off-site inspection days	0.5 (2.0)	0.5 (1.00)	0.83	-
Number of inspection protocols used	3.9 (2.7)	8.8 (3.6)	<0.001	1.70

LTCH, long-term care home; FNH, food nutrition and hydration; SD, standard deviation. Independent samples t-test. Levene’s test indicated unequal variances for number of inspection protocols used and off-site inspection days; Welch’s t-test was applied. Effect size reported as Cohen’s d (d), with interpretation: 0.20 = small, 0.50 = medium, 0.80 = large [41,42].

**Table 3:** Comparison of categorical LTCH and inspection characteristics with FNH non-compliance in Ontario Long-Term Care Homes in 2024 (*n* = 623).

Categorical Characteristics		No FNH Non-Compliance *n* = 547 *n* (%)	≥1 FNH Non-Compliance *n* = 76 *n* (%)	*p*-Value	Effect Size (Φ)
LTCH Characteristics					
Accreditation	Yes, 512 (82.2)	447 (81.7)	65 (85.5)	0.42	-
	No, 111 (17.8)	100 (18.3)	11 (14.5)		
Home ownership	For-profit, 343 (55.1)	306 (55.9)	37 (48.7)	0.04	0.10
	Non-profit, 173 (27.8)	143 (26.1)	30 (39.5)		
	Municipal, 107 (17.2)	98 (17.9)	9 (11.8)		
Region	Urban, 448 (71.9)	391 (71.5)	57 (75.0)	0.52	-
	Rural, 175 (28.1)	156 (28.5)	19 (25.0)		
Inspection Characteristics					
Complaint inspection	Yes, 231 (37.1)	204 (37.3)	27 (35.5)	0.77	-
	No, 392 (62.9)	343 (62.7)	49 (64.5)		
Critical Incident inspection	Yes, 470 (75.4%)	437 (79.9)	33 (43.4) ^b^	<0.001	0.28
	No, 153 (24.6%)	110 (20.1) ^b^	43 (56.6) ^a^		
Follow-Up inspection	Yes, 119 (19.1)	108 (19.7)	11 (14.5)	0.27	-
	No, 504 (80.9)	439 (80.3)	65 (85.5)		
PCI	Yes, 70 (11.2)	34 (6.2) ^b^	40 (52.6) ^a^	<0.001	0.43
	No, 553 (88.8)	513 (93.8)	36 (47.4) ^b^		
Other type of inspection	Yes, 24 (3.9)	17 (3.1)	7 (9.2) ^a^	0.01	0.10
	No, 599 (96.1)	530 (96.9)	69 (90.8)		
FNH Protocol	Yes, 151 (24.2)	85 (15.5) ^b^	66 (86.8) ^a^	<0.001	0.55
	No, 472 (75.8)	462 (84.5) ^a^	10 (13.2) ^b^		
Season of inspection	Spring, 147 (23.6)	131 (23.9)	16 (21.1)	0.63	-
	Summer, 159 (25.5)	141 (25.8)	18 (23.7)		
	Fall, 203 (32.6)	179 (32.7)	24 (31.6)		
	Winter, 114 (18.3)	96 (17.6)	18 (23.7)		

^a^ Higher-than-expected frequency, standardized residual > 1.96; ^b^ Lower-than-expected frequency, standardized residual < −1.96. LTCH, long-term care home; PCI, proactive compliance inspection; FNH, food, nutrition and hydration. Pearson Chi-Square. Effect size reported as Phi (Φ), with interpretation: 0.10 = small, 0.30 = medium, 0.50 = large [41,42]. Inspection types are not mutually exclusive and can occur in any combination; thus, the totals across Complaint, Critical Incident, Follow-up, and PCI exceed 100%.

## Data Availability

The database created for this study is currently being analyzed for further research; therefore, this database is not available at the present time. All inspection reports used for this study are currently publicly available on the Ministry of Long-Term Care public reporting website; however, ongoing discussions regarding limiting public access to these reports may limit our ability to share them in the future. To support transparency and reproducibility, the coding procedure is available from the authors upon request.

## Supplementary Materials

The following supporting information can be downloaded at: https://www.mdpi.com/article/10.3390/ijerph22111619/s1, Table S1: Sections and sub-sections of the Fixing Long-Term Care Act [5] and Ontario Regulation 246/22 [6] where non-compliances were found organized into categories; Table S2: Ministry of Long-Term Care Inspection Protocols used during study period (2024).

## Abbreviations

The following abbreviations are used in this manuscript:

LTCH	Long-Term Care Home
LTC	Long-Term Care
PCI	Proactive Compliance Inspection
FNH	Food, Nutrition and Hydration
