# Supplementary material for: What Gets Measured Gets Counted: Food, Nutrition, and Hydration Non-Compliance in Ontario Long-Term Care Homes and the Role of Proactive Compliance Inspections, 2024
Source: Int J Environ Res Public Health. 2025 Oct 23;22(11):1619. doi: 10.3390/ijerph22111619 (PMC12652318; doi:10.3390/ijerph22111619)
Supplement: Supplementary file 1 [file ijerph-22-01619-s001.zip › ijerph-3820692-supplementary.pdf]

Table S1. Sections and sub-sections of the Fixing Long-Term Care Act, 2021 [5] and Ontario Regulation 246/22 [6], where non-compliances were found, organized into categories.

Categories	Sections/sub-sections (n)
Administration	33
Staffing and Training	24
Food, Nutrition, and Hydration	20
Drugs and Medication Management	23
Facility Management	25
Infection Prevention and Control	13
Plan of Care	15
Prevention of Abuse and Neglect	17
Reporting and complaints	12
Required programs	13
Resident Care and Support Services	22
Residents' Rights and Representation	16
Responsive Behaviors	6
**Total**	239

Table S2. Ministry of Long-Term Care Inspection Protocols used during study period (2024).

Inspection Protocols (n=23)
Admissions, absences, and discharge
Continence care
Falls prevention and management
Food, nutrition and hydration
Housekeeping, laundry, and maintenance services
Infection prevention and control
Medication management
Pain management
Prevention of abuse and neglect
Quality improvements
Recreation and social activities
Reporting and complaints
Resident care and support services
Resident charges and trust accounts
Residents' and Family Councils
Residents' rights and choices
Responsive behaviors
Restraints / Personal Assistance Services Devices (PASD) management
Safe and secure home
Skin and wound prevention management
Staffing, training and care standards
Whistle-blowing protection and retaliation
Palliative care
